# Ingredients for robustness

**DOI:** 10.1007/s12064-020-00332-4

**Published:** 2020-12-02

**Authors:** Nihat Ay

**Affiliations:** 1 Max Planck Institute for Mathematics in the Sciences, Leipzig, Germany; 2 Leipzig University, Leipzig, Germany; 3 Santa Fe Institute, Santa Fe, NM USA

**Keywords:** Robustness, Knockouts, Neutrality, Interaction order

## Abstract

A core property of robust systems is given by the invariance of their function against the removal of some of their structural components. This intuition has been formalised in the context of input–output maps, thereby introducing the notion of exclusion independence. We review work on how this formalisation allows us to derive characterisation theorems that provide a basis for the design of robust systems.

## Introduction: What are robust systems?

**Fig. 1:**
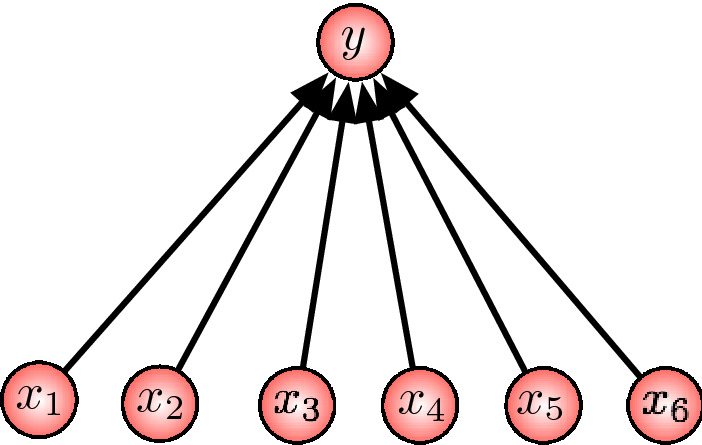
An input–output map for the study of robustness

The robustness of a system, be it biological or artificial, always refers to a set of perturbations. A biological system has to cope with a number of perturbations, including a constantly changing environment (Wagner 2007 gives a general introduction to the robustness of biological systems). One way to do so is by shaping and controlling its environment in a way that reduces perturbations. This is often referred to as niche construction, which can take place in the ecological as well as the social domain (Flack et al. 2006; Krakauer et al. 2009). Another way to deal with perturbations is by an intrinsic adaptation, change of the system itself and not the environment, so that the system’s function remains unchanged. It is this second way of coping with perturbations that we are addressing in this article. The setting in which we want to study this kind of robustness is kept very simple, as shown in Fig. 1. It is given by a (stochastic) map that receives *n* inputs and generates a, potentially high-dimensional, output. It is surprising how far-reaching this minimalistic setting is. It is general enough for studying the robustness of a number of biologically relevant mappings, such as the genotype-phenotype map, the genetic code, and neurons. However, addressing such application fields is beyond the scope of this article. Our aim is rather moderate. We review previous work on this subject and highlight the main insights in a more direct, instructive and conceptually complete way. The presented theory has been initially proposed by Ay and Krakauer (2007) and further developed in a number of works, including (Ay et al. 2007; Krakauer et al. 2010; Boldhaus et al. 2010; Rauh 2013; Rauh and Ay 2014). It touches upon important subjects related to robustness, such as niche construction, adaptation and neutrality, which have been addressed from various perspectives in a large number of publications. This article is by no means complete in providing a review of these publications. Instead, it restricts attention to the mentioned works in which connections to other works are outlined more thoroughly.

In order to study edge or node deletion, which we also call *knockout intervention* or simply *knockout*, we consider a single unit of the network, the output unit, together with all its parents $I := \{1,\ldots ,n\}$, which provide the input. We denote the input variables by $X_1, \ldots , X_n$ and the output variable by *Y*. Correspondingly, the values of $X_i$ are denoted by $x_i$ and the values of *Y* by *y* (see Fig. 1). Throughout this article, we assume that $x_i$ and *y* are elements of some non-empty and finite state spaces, ${\mathcal {X}}_i$ and ${\mathcal {Y}}$, respectively. For a subset *J* of *I*, we write ${\mathcal {X}}_J$ for the Cartesian product of the ${\mathcal {X}}_i$, $i \in J$, and denote its elements by $\varvec{x}_J$.

**Fig. 2:**
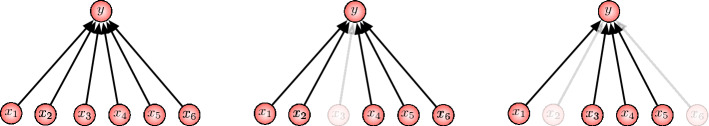
Left: unperturbed function. Middle: knockout of node 3. Right: knockout of nodes 2 and 6

We model the mechanism of *Y* as a stochastic map $\kappa (\varvec{x};y)$ which assigns to each input $\varvec{x} = (x_1,\ldots , x_n)$ a random output *y*, that is $\kappa (\varvec{x};y) \ge 0$ and $\sum _{y'} \kappa (\varvec{x} ; y') = 1$ for all $\varvec{x}$, *y*. Such a map is called a *Markov kernel*. In this article, we study robustness with respect to knockouts of some of the input nodes *I*. When a subset *K* of the input nodes is removed, typically the behaviour of the remaining system with input nodes $J = I {\setminus } K$ will be different. Without further assumptions, the post-knockout function is not determined by $\kappa$ and can be anything. We therefore consider another stochastic map, a Markov kernel, $\kappa _J: {\mathcal {X}}_{J} \times {\mathcal {Y}} \rightarrow [0,1]$ as model of the post-knockout function (see Fig. 2).

A complete specification of the function is given by the family ${(\kappa _J)}_{J \subseteq I}$ of all possible post-knockout functions, which we refer to as *functional modalities*. The Markov kernel $\kappa$ itself, which describes the behaviour of the system prior to any knockouts, can be identified with $\kappa _{I}$. Let us now come back to robustness. Intuitively, a system is considered to be robust if its post-knockout function coincides with, or does not deviate too much from, its pre-knockout function. Clearly, this cannot be non-trivially achieved if it has to hold for all knockout perturbations. However, if we upper bound the number of deleted input nodes, typically by a much smaller number than *n*, then the problem of characterising robust systems turns out to be quite interesting. We will review such characterisations in this article.

In “Exclusion dependence” section, we provide a quantitative approach to the outlined invariance of function with respect to knockouts, referred to as *exclusion dependence*. Basically, it measures the deviation of the post-knockout function from the pre-knockout function. This already allows us to present our first characterisation of robust systems in “Neutrality” section, which is related to *neutrality*. In “Mechanistic modelling of knockout interventions” section, we introduce a mechanistic description of knockout perturbations, thereby relying on notions from statistical physics. This allows us to solve the problem of system identification based on experimental knockouts in terms of the Möbius inversion. The solution is closely related to the Gibbs–Markov equivalence known from statistical physics. We present the subject of system identification in “Knockout interventions for system identification” section. Intuitively, the difficulty of a system’s identification in terms of knockout experiments is directly correlated with the robustness of that system. This follows formally from our second characterisation of robust systems, presented in “Robustness and interaction order” section. In the “Conclusions” section, we compare the two presented descriptions of robust systems and outline directions of research.

## Exclusion dependence

By exclusion dependence, we mean the deviation or distance of the post-knockout function from the original function. With an appropriate distance measure, knockout interventions can be studied in a quantitative manner, which allows us to derive a general robustness measure. Conceptually, any measure of robustness should incorporate the invariance of the system’s function against knockout of input nodes, say the nodes of a subset *K*. After the knockout, the system will have access only to the remaining input nodes, those in the complement $J := I {\setminus } K$. In general, the function will be affected by such a knockout intervention. We measure the deviation of the post-knockout function $\kappa _J$ from the original function $\kappa$ in $\varvec{x} = (\varvec{x}_J , \varvec{x}_K)$ by using the relative entropy or Kullback-Leibler divergence (KL-divergence) as a distance measure. For two probability vectors *p* and *q* on a finite set ${\mathcal Z}$, it is defined as

$$\begin{aligned} D(p \, \Vert \, q) := \sum _{z \in {\mathcal Z}} p(z) \ln \frac{p(z)}{q(z)}, \end{aligned}$$

with the convention $p(z) \ln \frac{p(z)}{q(z)} = 0$ if $p(z) = 0$, and $p(z) \ln \frac{p(z)}{q(z)} = \infty$ if $p(z) > q(z) = 0$. The KL-divergence satisfies the following fundamental property:

1 $$\begin{aligned} D(p\Vert q) \ge 0, \,\,\, \text{ and } \,\,\, D(p\Vert q) = 0 \;\; \text{ if } \text{ and } \text{ only } \text{ if } \;\; p = q. \end{aligned}$$

The KL-divergence is well-known as a canonical divergence in information geometry (Amari and Nagaoka 2000; Amari 2016; Ay and Amari 2015; Ay et al. 2017). We now use the KL-divergence in order to quantify the deviation of the post-knockout function $\kappa _J$ from the original function $\kappa$, given that they both have input $\varvec{x} = (\varvec{x}_J, \varvec{x}_K)$:

2 $$\begin{aligned} D\big ( \kappa (\varvec{x}_J , \varvec{x}_K; \cdot ) \, \Vert \, \kappa _J (\varvec{x}_J ; \cdot ) \big ) := \sum _{y} \kappa (\varvec{x}_J , \varvec{x}_K ; y) \ln \frac{\kappa (\varvec{x}_J , \varvec{x}_K ; y)}{\kappa _J ( \varvec{x}_J ; y)}. \end{aligned}$$

Given the property (1) of the KL-divergence, this deviation vanishes if and only if the post-knockout function coincides with the original function in $\varvec{x} = (\varvec{x}_J, \varvec{x}_K)$, that is

3 $$\begin{aligned} \kappa (\varvec{x}_J , \varvec{x}_K ; y) = \kappa _J ( \varvec{x}_J ; y) \qquad \text{ for } \text{ all } y. \end{aligned}$$

Note that this invariance of the function refers to the input $\varvec{x}$. This does not necessarily mean that the function will also be invariant in the context of another input $\varvec{x}'$. Let us consider an input distribution $\mu$ with support ${\mathcal {S}}$. Taking the mean of the individual KL-divergences (2) with respect to $\mu$ yields

$$\begin{aligned} D_{\mu } (\kappa \, \Vert \, \kappa _J) \, := \, \sum _{\varvec{x}} \mu (\varvec{x}) \sum _{y} \kappa (\varvec{x} ; y) \ln \frac{\kappa ( \varvec{x} ; y)}{\kappa _J ( \varvec{x}_J ; y)} , \end{aligned}$$

which we refer to as *exclusion dependence* (Ay and Krakauer 2007). Obviously, the exclusion dependence vanishes if and only if the invariance (3) holds for all $\varvec{x} \in {\mathcal {S}}$. We now relate the invariance of function to stochastic independence. To be more precise, we set

$$\begin{aligned} p(\varvec{x}_J, \varvec{x}_K, y):= & {}\, \mu (\varvec{x}_J, \varvec{x}_K) \, \kappa (\varvec{x}_J, \varvec{x}_K ; y) \\= & {} \,\mathrm{Prob}\left\{ X_J = \varvec{x}_J, X_K = \varvec{x}_K, Y = y \right\} \end{aligned}$$

and define the *conditional mutual information* of *Y* and $X_K$, given $X_J$:

$$\begin{aligned}&I(Y ; X_{K} \, | \, X_J)\\&\quad := \sum _{\varvec{x}_J} p(\varvec{x}_J) \sum _{y, \, \varvec{x}_K} p(y , \varvec{x}_K | \varvec{x}_J) \ln \frac{p(y , \varvec{x}_K | \varvec{x}_J)}{p(y | \varvec{x}_J) \, p(\varvec{x}_K| \varvec{x}_J)} \\&\quad = \sum _{\varvec{x}_J,\, \varvec{x}_K} p(\varvec{x}_J, \varvec{x}_K) \sum _{y} p(y | \varvec{x}_J , \varvec{x}_K) \ln \frac{p(y | \varvec{x}_J , \varvec{x}_K)}{p(y | \varvec{x}_J)} . \end{aligned}$$

Instead of considering the actual post-knockout function $\kappa _J$, we now ask how close we can be in principle to the original function, if we allow any post-knockout function $\kappa _J'$ from the set ${\mathcal K}_J$ of Markov kernels that only use the remaining nodes *J* as input units. Clearly, the actual post-knockout function $\kappa _J$ is contained in that set, which gives us the following inequality:

4 $$\begin{aligned}&D_{\mu } (\kappa \, \Vert \, \kappa _J) \; \ge \; \inf _{\kappa _J' \in {\mathcal K}_J} D_{\mu } (\kappa \, \Vert \, \kappa _J') \nonumber \\&\quad = \; D_{\mu }(\kappa \, \Vert \, \kappa _J^*) \; = \; I(Y ; X_{K} \, | \, X_J) \; \ge \; 0. \end{aligned}$$

**Fig. 3:**
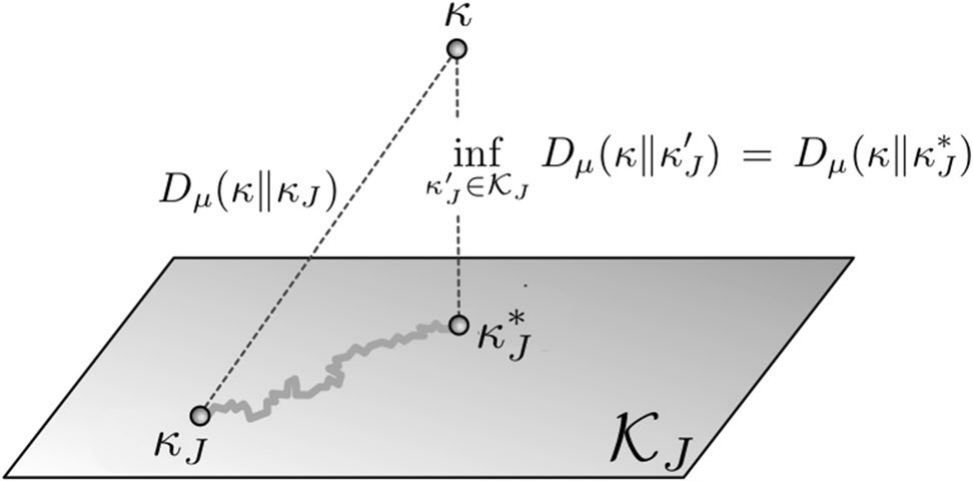
Optimal function after knockout

Here, $\kappa _J^*$ is chosen such that, among all post-knockout functions, it has the smallest achievable deviation from the original function. The geometric interpretation of this minimisation is shown in Fig. 3.

One can show that any such minimiser has to satisfy the following condition.

5 $$\begin{aligned} \kappa _J^*( \varvec{x}_J ; y) \, = \, p(y | \varvec{x}_J), \qquad \text{ whenever } p(\varvec{x}_J) > 0. \end{aligned}$$

Thus, the post-knockout function is almost uniquely determined. If the exclusion dependence vanishes then, with the estimate (4), we have:

1. The conditional mutual information $I(Y ; X_{K} \, | \, X_J)$ vanishes. This is equivalent to the conditional independence $Y \perp \!\!\!\perp X_{K} \, | \, X_J$. This is a property of $\mu$ and $\kappa$ together.
2. The post-knockout function $\kappa _J$ is a minimiser of the exclusion dependence, so that it is (almost) uniquely determined by the RHS of (5).

In what follows, we want to require exclusion independence for a number of knockout scenarios, not just restricted to one subset *K*. Assume that we want the function to be invariant with respect to the deletion of at most *k* input units. In that case, we have exclusion independence for all *K* with $|K| \le k$. Thus,

6 $$\begin{aligned} Y \perp \!\!\!\perp X_{ K } \, | \, X_{I {\setminus } K} \,\,\,\text{ for } \text{ all } \,K \subseteq I, |K| \le k. \end{aligned}$$

These conditional independence statements encode in an implicit way those probability distributions that ensure exclusion independence. Again, these conditions refer to the input distribution $\mu$ and the Markov kernel $\kappa$. The family of post-knockout functions $\kappa _{I {\setminus } K}$, $|K| \le k$, is then (almost) uniquely determined by $\mu$ and $\kappa$. Using algebraic geometric methods, one can determine those pairs $\mu$ and $\kappa$ that satisfy Eq. (6) (Rauh 2013). An outcome of this analysis is Theorem 1 presented below. It highlights the role of neutrality for robust systems. To be more precise, assume that we have exclusion independence against the knockout of any node set *K* with at most *k* elements. Then, for two configurations $(\varvec{x}_J, \varvec{x}_K)$ and $(\varvec{x}_J, \varvec{x}'_K)$ in ${\mathcal {S}}$, we have

7 $$\begin{aligned} \kappa (\varvec{x}_J, \varvec{x}_K ; y)= & {} \,p(y | \varvec{x}_J, \varvec{x}_K) \, = \, p(y | \varvec{x}_J)\nonumber \\= & {}\, p(y | \varvec{x}_J, \varvec{x}'_K) \, = \, \kappa (\varvec{x}_J, \varvec{x}'_K ; y). \end{aligned}$$

Note that this property refers to $\kappa$ and does not involve the post-knockout function $\kappa _J$. It basically says that any function $\kappa$ that is invariant with respect to knockouts has also to be invariant with respect to mutations (change of the states of *K* from $\varvec{x}_K$ to $\varvec{x}_K'$). Mutational neutrality has been the subject of genetic robustness (Arjan et al. 2003; Schuster et al. 1994; Fontana 2006). We are now going to highlight neutrality from a geometric perspective.

## Neutrality

Both, the input distribution $\mu$ as well as the Markov kernel $\kappa$, are involved in the condition (6). We now present a way how to construct $\kappa$ so that the condition (6) is satisfied, based on a given input distribution $\mu$. Let us assume that we want to delete at most *k* input nodes while keeping the function (stochastic map) invariant. The Hamming distance of two input configurations $\varvec{x} = (x_1,\ldots ,x_n)$ and $\varvec{x}' = (x_1', \ldots , x_n')$ is given by the number of indices *i* for which $x_i \not = x_i'$. We now connect two configurations $\varvec{x}$ and $\varvec{x}'$ in the support ${\mathcal {S}}$ of $\mu$ if their Hamming distance is smaller than or equal to *k*. This way, we obtain a graph with node set ${\mathcal {S}}$. The connected components of this graph play a particularly important role for exclusion independence. It turns out that exclusion independence is achieved by the stochastic map $\kappa$, which takes an input configuration from ${\mathcal {X}}_I$ and generates a stochastic output $y \in {\mathcal {Y}}$, if and only if it is neutral on these connected components. More precisely, if $\kappa (\varvec{x}; y) = \kappa (\varvec{x}'; y)$ for all $y \in {\mathcal {Y}}$ whenever $\varvec{x}$ and $\varvec{x}'$ are from the same connected component, then the condition (6) is satisfied. The values of the Markov kernel outside of ${\mathcal {S}}$ do not play a role here.

**Fig. 4:**
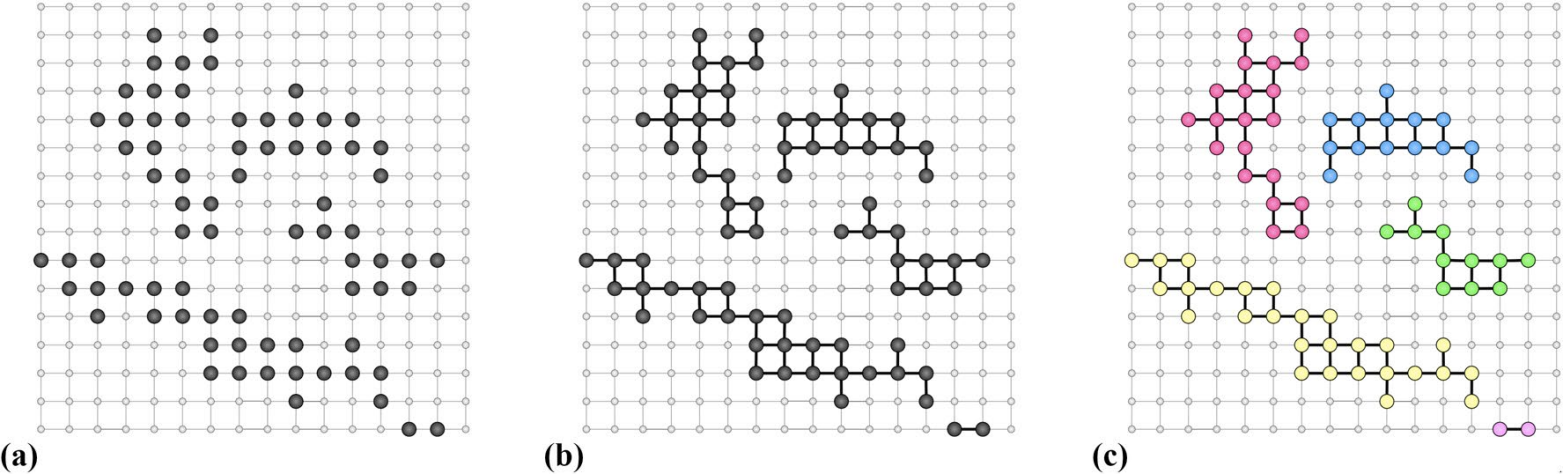
**a** A set ${\mathcal {S}}$ in ${\mathcal {X}}_I$; **b** the connected components of ${\mathcal {S}}$; **c** neutrality of the map on the connected components, indicated by colour

In order to illustrate the construction, let us consider an example. Say that we have only two input units with state sets ${\mathcal {X}}_1 = {\mathcal {X}}_2 = \{1,\ldots , 16\}$, and assume that out of the 256 joint configurations, some of them are in the support ${\mathcal {S}}$ of the input distribution $\mu$, as shown in Fig. 4a.

For the illustration of the construction, it is easier to consider the graph metric given by the network shown in Fig. 4 a, instead of the Hamming distance. This yields the induced network where two elements in ${\mathcal {S}}$ are connected if their distance equals one. The resulting connected components are shown in Fig. 4b. We denote the set of connected components *N* by $\mathfrak {N}({\mathcal {S}})$. Now we are free to define $\kappa$ in such a way that it outputs the same distribution $\lambda _N (y)$ on ${\mathcal {Y}}$ for two elements $\varvec{x}$, $\varvec{x}'$ contained in the same connected component *N*. This neutrality is illustrated by colouring each of the connected components by a single colour in Fig. 4c.

### Theorem 1

(see Proposition 3 in Rauh and Ay 2014) *Let* ${\mathcal {S}}$ *be a non-empty set of input configurations. We connect two points in* ${\mathcal {S}}$ *if their Hamming distance is at most* *k*. *The resulting network divides* ${\mathcal {S}}$ *into connected components, and we denote their set by* $\mathfrak {N}({\mathcal {S}})$. *Furthermore, consider a probability measure* $\alpha$ *on* $\mathfrak {N}({\mathcal {S}})$, *and for each* $N \in \mathfrak {N}({\mathcal {S}})$ *a probability measure* $\mu _N$ *on* ${\mathcal {X}}_{I}$ *with support* *N* *and a probability measure* $\lambda _N$ on ${\mathcal {Y}}$. *Then the following joint distribution satisfies the conditional independence statements* (6):

8 $$\begin{aligned} p(\varvec{x} , y) \; = \; \sum _{N \in \mathfrak {N}(S)} \alpha (N) \, \mu _N(\varvec{x}) \, \lambda _N(y). \end{aligned}$$

*On the other hand, any joint distribution that satisfies the conditional independence statements* (6) *has the structure* (8).

**Fig. 5:**
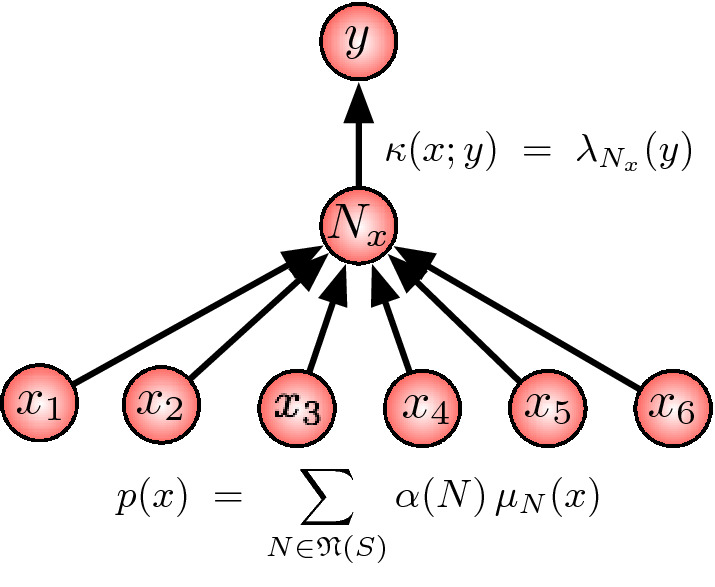
Structure of a robust map

The situation is illustrated in Fig. 5. The result of Theorem 1 highlights the concept of neutrality which is an important concept in robustness studies (Wagner 2007).

Given an input distribution $\mu$, this theorem allows us to construct stochastic maps $\kappa$ that ensure *k*-exclusion independence. This is done in several steps.

1. Consider the connected components $\mathfrak {N}({\mathcal {S}})$ of the support set ${\mathcal {S}}$ of $\mu$.
2. For each $N \in \mathfrak {N}({\mathcal {S}})$, choose a probability distribution $\lambda _N$ on ${\mathcal {Y}}$.
3. For $\varvec{x} \in {\mathcal {S}}$, we denote by $N_{\varvec{x}}$ the connected component $N \in \mathfrak {N}({\mathcal {S}})$ that contains $\varvec{x}$ and define
  $$\begin{aligned} \kappa (\varvec{x} ; y) \, := \, \lambda _{N_{\varvec{x}}}(y). \end{aligned}$$
  Outside of ${\mathcal {S}}$, $\kappa$ can be chosen arbitrarily.

## Mechanistic modelling of knockout interventions

In what follows, we introduce a particular way of specifying a family $(\kappa _J)$ of functional modalities. We consider a function $Q(\varvec{x};y)$ that describes the affinity of the node to assume the state *y* given the input $\varvec{x} = (x_1, \ldots , x_n)$. With such a function, the corresponding Markov kernel can be defined as

9 $$\begin{aligned} \kappa (\varvec{x}; y) = \frac{e^{Q(\varvec{x};y)}}{\sum _{y'} e^{Q(\varvec{x};y')}} . \end{aligned}$$

Here, we adopt the terminology from statistical physics, where the structure (9) of a so-called *Boltzmann–Gibbs distribution* originates from, and refer to the function *Q* also as an *energy function*. Without any further specification of the energy function, the structure (9) of a stochastic map is quite general. The only restriction comes from the fact that all transition probabilities $\kappa (\varvec{x};y)$ are positive. This implies, for instance, that deterministic functions, such as Boolean functions, cannot be modelled in this way directly. However, these functions can be approximated in terms of stochastic maps of the form (9) arbitrarily well.

One can interpret the structure (9) as a “black-box” description of a stochastic input–output map, which does not incorporate any information about the mechanisms that actually give rise to that map. In fact, one can imagine many mechanisms that generate the same input–output mapping. In general, each of these mechanisms will lead to a different function as result of a deletion of input units. Therefore, the prediction of a post-knockout function requires some level of mechanistic description. We now introduce such a description and demonstrate how it can be used to predict any post-knockout function of the system. The main idea is to decompose the energy function *Q* into interaction terms $\phi _A(\varvec{x}_A; y)$, thereby employing another concept from statistical physics. Each of these terms quantifies to what extent the input nodes in *A* work together and thereby contribute to the overall function. Their contribution can be positive or negative, that is excitatory or inhibitory.

**Fig. 6:**
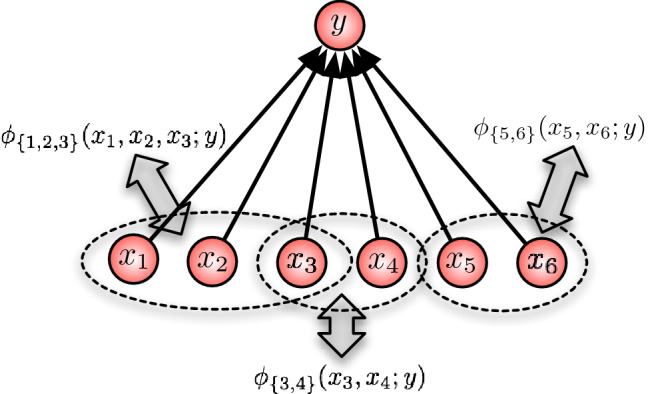
Decomposition of the affinity

As an illustration, consider the example shown in Fig. 6. In this example, there are three interaction terms that contribute to the function *Q* and thereby to the output *y* via the stochastic map (9):

10 $$\begin{aligned}&Q(x_1,\ldots , x_6 ; y) \, = \, \phi _{\{1,2,3\}}(x_1,x_2,x_3 ; y) \nonumber \\&\quad + \phi _{\{3, 4\}}(x_3,x_4 ; y) + \phi _{\{5, 6\}}(x_5,x_6 ; y). \end{aligned}$$

**Fig. 7:**
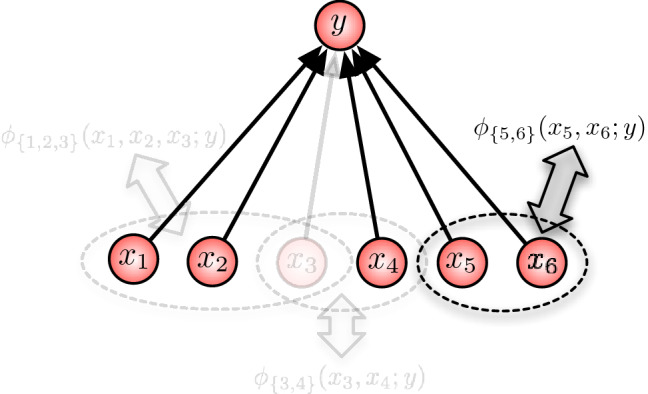
Removal of the interaction terms $\phi _{\{1,2,3\}}(x_1,x_2,x_3 ; y)$ and $\phi _{\{3, 4\}}(x_3,x_4 ; y)$ as result of the knockout of node 3

This additional information on how the function *Q* is composed in individual interaction terms already allows us to model and study knockouts. To illustrate this, let us assume that, for instance, node 3 is knocked out. As it is not present anymore, it cannot interact with any other node. Therefore, it is natural to assume that, as result of the knock-out intervention, all interactions $\phi _A(x_A;y)$ that involve node 3 are now discarded. In our example, this corresponds to removing $\phi _{\{1,2,3\}}(x_1,x_2,x_3 ; y)$ and $\phi _{\{3, 4\}}(x_3,x_4 ; y)$ from the right-hand side of (10), as illustrated in Fig. 7. With $J := \{1,2,4,5,6\}$— node 3 is removed—the resulting post-knockout function is then given as

11 $$\begin{aligned} \kappa _{J}(\varvec{x}_J ; y) \, = \, \frac{e^{\phi _{\{5,6\}}(x_5,x_6;y)}}{\sum _{y'} e^{\phi _{\{5,6\}}(x_5,x_6;y')}}. \end{aligned}$$

Note that this is a modification of the stochastic map (9), where all contributions of node three are removed from the energy function *Q*. Let us discuss two situations here. Let us first assume that the post-knockout function is different from the original function. This implies that node 3 is involved in at least one interaction term, so that we would be able to infer some mechanistic aspects of the role that node 3 plays in generating the output *y*. However, we cannot conclude that node 3 does not play any role if we do not see a difference between the pre- and post-knockout functions. This invariance of function is an essential requirement for robustness. A robust system, therefore, does not easily reveal its internal mechanisms as a result of knockout interventions. Our simple example already highlights two roles of knockout interventions or perturbations: We can distinguish between experimental knockouts, with which one aims at understanding the inner working of a system, and knockouts as exogenous perturbations that the system has to compensate in order to perform its function. The subjects of system identification and robustness of systems will be discussed in further detail in the following sections. However, let us first extend our instructive example to the general setting of an arbitrary number of input nodes and a general interaction structure.

Assume that the energy function *Q* can be decomposed in terms of interactions $\phi _A$, $A \subseteq I$, that is

12 $$\begin{aligned} Q(\varvec{x}; y) \; = \; \sum _{A \subseteq I} \phi _A(\varvec{x}_A;y), \end{aligned}$$

which corresponds to (10). Consider the situation where a number of input nodes is knocked out and denote the set of remaining nodes by *J*. Then each interaction term that involves a node from the complement of *J* will be removed from the decomposition (12), leading to the direct extension of (11)

13 $$\begin{aligned} \kappa _J(\varvec{x}_J; y)= & {} \frac{1}{Z(\varvec{x}_J)} \, \exp \left\{ {\sum _{A \subseteq J} \phi _A( \varvec{x}_A; y)} \right\} , \qquad J \subseteq I, \end{aligned}$$

14 $$\begin{aligned} \text{ with } \qquad Z(\varvec{x}_J)= & {} \sum _{y'} \exp \left\{ {\sum _{A \subseteq J} \phi _A(\varvec{x}_A; y')}\right\} . \end{aligned}$$

This family of stochastic maps, indexed by the subsets *J* of remaining nodes, describes all post-knockout functions, which represents one instance of the *functional modalities* we discussed above. As we will see, this is a quite general mechanistic perspective in the sense that any family of strictly positive functional modalities can be represented this way.

Let us further illustrate this approach to modelling post-knockout functions in terms of the following example from the field of neural networks.

### Example 1

We consider a neuron which receives an input $\varvec{x} = (x_1,\ldots ,x_n) \in {\{-1,+1\}}^n$ and generates the output $+1$ with probability

15 $$\begin{aligned} \kappa (\varvec{x} ; +1) \; := \; \frac{1}{1 + e^{-\sum _{i = 1}^n w_i \, x_i}}. \end{aligned}$$

For a general output $y \in \{- 1, + 1\}$, this implies

16 $$\begin{aligned} \kappa (\varvec{x} ; y) \; := \; \frac{ e^{\frac{1}{2} \sum _{i = 1}^n w_i \, x_i \, y }}{e^{\frac{1}{2} \sum _{i = 1}^n w_i \, x_i \cdot (-1)} + e^{ \frac{1}{2} \sum _{i = 1}^n w_i \, x_i \cdot ( + 1 )}} \, . \end{aligned}$$

This representation of the stochastic map $\kappa$ has a structure that allows us to infer the function after a knockout of a set *K* of input nodes, by simply removing the contribution of all the nodes in *K*. This leads to

$$\begin{aligned} \kappa _J(\varvec{x}_J; +1) \; = \; \frac{1}{1 + e^{-\sum _{i \in J} w_i \, x_i}}, \end{aligned}$$

where $J = I {\setminus } K$. This inference of the post-knockout function is based on the decomposition of the sum that appears in the enumerator on the RHS of (16), $\frac{1}{2} \sum _{i = 1}^n w_i \, x_i \, y$. Mapping this to the representation (12), we clearly have $\phi _A = 0$ whenever $|A| \not = 1$, and $\phi _{\{i\}}(x_i ; y) = \frac{1}{2} \, w_i \, x_i \, y$, $i = 1,\ldots ,n$.

## Knockout interventions for system identification

If the removal of some part of the system causes the loss or disturbance of one of its functions, then it is reasonable to attribute that function to the removed part. This intuition underlies, for instance, some of our understanding of brain function which has been deduced from brain lesions. Such lesions are typically caused by an injury or disease. However, the same fundamental intuition also underlies knockout experiments that aim at the identification of the system’s mechanisms. One well-known example is given by gene knockouts.

Let us now study this intuition based on our formal model of knockout interventions which we developed in “Mechanistic modelling of knockout interventions” section. We begin with the example shown in Fig. 6. We have already discussed the knockout of node 3, which gave us the post-knockout function (11). This implies

17 $$\begin{aligned}&\ln \kappa _{\{1,2,4,5,6\}}(x_1,x_2, x_4, x_5, x_6; y)\nonumber \\&\quad = Z(x_1,x_2,x_4,x_5,x_6) + \phi _{\{5,6\}}(x_5, x_6; y). \end{aligned}$$

If we knock out nodes 4 and 5, the remaining contribution is from the nodes 1, 2, and 3:

18 $$\begin{aligned}&\ln \kappa _{\{1,2,3\}}(x_1, x_2,x_3; y) \nonumber \\&\quad = Z(x_1,x_2,x_3) + \phi _{\{1,2,3\}}(x_1, x_2, x_3; y). \end{aligned}$$

Finally, for the knockout of nodes 1 and 5, the remaining contribution comes from nodes 3 and 4:

19 $$\begin{aligned}&\ln \kappa _{\{3,4\}}(x_3, x_4; y) \nonumber \\&\quad = Z(x_3,x_4) + \phi _{\{3,4\}}(x_3, x_4; y). \end{aligned}$$

This shows that we can recover the interactions, up to some function of $\varvec{x}$, given by the normalisations *Z*, if we know the post-knockout function of some appropriately chosen knockouts. This backs up the strategy of system identification in terms of knockout experiments. The non-uniqueness of the recovered interaction terms, due to the functions that only depend on the input $\varvec{x}$, does not harm here. Any family of interaction terms will represent the functional modalities $\kappa _J$, $J \subseteq I$, equally well.

Note that the knock-outs that reveal the individual interaction terms are also not unique. There are several appropriate knockout protocols. For instance, in the above example, we could also consider the three knockouts of node 1, node 4, and node 5, leading to a linear system

$$\begin{aligned}&\ln \kappa _{\{2,3,4,5,6\}}(x_2, x_3, x_4, x_5, x_6; y) \\&\quad = Z(x_2,x_3, x_4, x_5, x_6) \\&\quad \quad +\,\phi _{\{3,4\}}(x_3, x_4 ; y) + \phi _{\{5,6\}}(x_5, x_6 ; y) , \\&\ln \kappa _{\{1,2,3,5,6\}}(x_1, x_2 , x_3, x_5, x_6; y)\\&\quad =\, Z(x_1, x_2, x_3, x_5, x_6) \\&\quad \quad +\,\phi _{\{1,2,3\}}(x_1, x_2, x_3 ; y) + \phi _{\{5,6\}}(x_5, x_6 ; y) , \\&\ln \kappa _{\{1,2,3,4,6\}}(x_1,x_2, x_3, x_4, x_6; y)\\&\quad =\,Z(x_1, x_2, x_3, x_4,x_6)\\&\quad \quad +\,\phi _{\{1,2,3\}}(x_1, x_2, x_3 ; y) + \phi _{\{3,4\}}(x_3, x_4 ; y). \end{aligned}$$

This can be easily solved, and we obtain again the interactions from the post-knockout functions. Altogether, we have shown that two different knockout protocols, each of them involving only three knockout interventions, allow us to identify the three interaction terms of the system. In general, the situation is much more complex. On the one hand, there can be many more interaction terms involved so that we will require correspondingly many knockout interventions. We cannot assume that performing these interventions will always be feasible. On the other hand, even if only a few interactions are actually involved, without prior knowledge on these interactions it is not possible to determine a correspondingly small set of knockouts that would allow us to reveal the interactions within the system. Let us assume, for the moment, that we can perform all possible knockouts and measure the corresponding post-knockout functions. In theory, the resulting set of equations can be solved in terms of the Möbius inversion. Let us be more precise: Clearly, each $\kappa _J$ in (13) is strictly positive. Using the Möbius inversion, it is easy to see that each strictly positive family $(\kappa _J)$ has such a representation. In order to see this, we simply set

20 $$\begin{aligned} \phi _A (\varvec{x}_A, y) \; := \; \sum _{J \subseteq A} (-1)^{| A {\setminus } J |} \ln \kappa _J(\varvec{x}_J ; y) \, . \end{aligned}$$

Note that this representation is not unique: If an arbitrary function of $\varvec{x}_{A}$ is added to the function $\phi _{A}$, then the functional modalities remain unchanged. We have a one-to-one correspondence, modulo this ambiguity, between functional modalities and interactions:

$$\begin{aligned} \kappa _J, \; J \subseteq I \,\longleftrightarrow \, \phi _A, \;A \subseteq I. \end{aligned}$$

In this section, we do not interpret a knockout as a natural perturbation but as an experimental test. The intention is to reveal the inner working, the mechanisms, of the system based on its response to these unnatural perturbations. Indeed, we can identify important aspects of the interactions. This is remarkable, because these interactions provide the basis of the unperturbed function. Thus, we perturb the system in order to understand the constituents of its unperturbed function. This provides a formal basis for knockout experiments.

It is important to note that the choice of the $\phi _A$, $A \subseteq I$, for describing knockouts is essential. If we choose to represent a post-knockout function in terms of different interactions, say $\psi _B$, $B \subseteq I$, we have to translate the effect of knockouts accordingly. To be more precise, assume that we can represent the initial functions as

21 $$\begin{aligned} \phi _A(\varvec{x}_A ; y) \, = \, \sum _{B \subseteq A} \alpha _{A, B} \, \psi _B (\varvec{x}_B ; y), \qquad A \subseteq I. \end{aligned}$$

This implies

22 $$\begin{aligned} \ln \kappa _J(\varvec{x}_J ; y)\sim & {} \sum _{A \subseteq J} \phi _A(\varvec{x}_A ; y) \qquad \qquad (\text{by } (13)) \end{aligned}$$

23 $$\begin{aligned}= & {} \sum _{A \subseteq J} \sum _{B \subseteq A} \alpha _{A, B} \, \psi _B (\varvec{x}_B ; y) \qquad \quad \;\;(\text{by } (21)) \nonumber \\= & {} \sum _{B \subseteq J} \left( \sum _{C \subseteq J {\setminus } B} \alpha _{B \cup C, B} \right) \, \psi _B (\varvec{x}_B ; y) \nonumber \\= & {} \sum _{B \subseteq J} \beta _{J,B} \, \psi _B (\varvec{x}_B ; y) , \end{aligned}$$

where we define the coefficients $\beta _{J,B}$ accordingly. This calculation highlights the following fact: When describing the post-knockout function $\kappa _J$ in terms of the $\phi _A$ we simply remove all terms $\phi _A$ for which *A* is not contained in *J*, as described by (22). This is the basic requirement that defines the $\phi _A$, $A \subseteq I$. When trying to represent the post-knockout function in terms of different interactions, $\psi _B$, $B \subseteq I$, this rule changes. In order to see this, let us analyse the representation (23). Also in this case, the terms $\psi _B$ do not appear whenever *B* is not contained in *J*. However, the coefficients $\beta _{J,B}$ change as a consequence of a knockout. That means, if we have an expansion of $\ln \kappa (\varvec{x} ; y)$, where $\kappa$ is the unperturbed function, into terms $\psi _B$, with weights $\beta _{I,A}$, then simply removing weighted terms as result of a knockout is not sufficient.

## Robustness and interaction order

Let $(\kappa _J)$ be functional modalities, which we assume to be strictly positive. We know that they can be represented in terms of interactions, by the choice (20). How is exclusion independence mapped to this representation? In order to answer this question, let us consider the situation where at most *k* input nodes are knocked out. In this case, *k*-exclusion independence implies that for $|J| \ge r := n - k$, $B \subset J$ with $|B| = r$, and $\varvec{x} \in {\mathcal {S}}$, we have the equality $\kappa _J(\varvec{x}_J ; \cdot ) = \kappa _{B}(\varvec{x}_{B}; \cdot )$. Below we use the Möbius inversion (20) for the derivation of the interaction terms $\phi _A (\varvec{x}_A ; y)$ under the assumption of *k*-exclusion independence. In order to do so, we need to evaluate the following sum:

$$\begin{aligned}&\sum _{\begin{array}{c} J \subseteq A \\ | J | \ge r \end{array}} (-1)^{| A {\setminus } J |} \ln \kappa _J (\varvec{x}_J ; y )\\&\quad = \sum _{\begin{array}{c} J \subseteq A \\ |J| \ge r \end{array}} (-1)^{| A {\setminus } J |} \, \frac{1}{{ \left( {\begin{array}{c}|J|\\ r\end{array}}\right) }} \sum _{\begin{array}{c} B \subseteq J \\ | B | = r \end{array}} \ln \kappa _J (\varvec{x}_J ; y ) \\&\quad = \sum _{\begin{array}{c} J \subseteq A \\ |J| \ge r \end{array}} (-1)^{| A {\setminus } J |} \, \frac{1}{{ \left( {\begin{array}{c}|J|\\ r\end{array}}\right) } } \sum _{\begin{array}{c} B \subseteq J \\ | B | = r \end{array}} \ln \kappa _B (\varvec{x}_B ; y ) \\&\quad = \sum _{\begin{array}{c} B \subseteq A \\ |B| = r \end{array}} \left\{ \sum _{R \subseteq A {\setminus } B } (-1)^{| A | - |R| - r} \, \frac{1}{{ \left( {\begin{array}{c}|R| + r\\ r\end{array}}\right) } } \right\} \, \ln \kappa _B ( \varvec{x}_B ; y ) . \end{aligned}$$

Together with (20) this gives us

24 $$\begin{aligned} \phi _A (\varvec{x}_A ; y)= & {} \sum _{\begin{array}{c} J \subseteq A \\ | J | < r \end{array}} (-1)^{| A {\setminus } J |} \ln \kappa _J(\varvec{x}_J ; y) \nonumber \\&+\sum _{\begin{array}{c} J \subseteq A \\ | J | \ge r \end{array}} (-1)^{| A {\setminus } J |} \ln \kappa _J(\varvec{x}_J ; y) \nonumber \\= & {} \sum _{\begin{array}{c} J \subseteq A \\ | J | \le r \end{array}} \alpha _{A,J} \, \ln \kappa _J(\varvec{x}_J ; y) , \end{aligned}$$

where

$$\begin{aligned} \alpha _{A,J} = {\left\{ \begin{array}{ll} (-1)^{|A| - |J|}, &{} \text { if } |J|< r \\ \sum _{R \subseteq A {\setminus } J } (-1)^{| A | - |R| - r} \, \frac{1}{{ \left( {\begin{array}{c}|R| + r\\ r\end{array}}\right) } },&\text { if } |J|= r \end{array}\right. } \end{aligned}$$

depends only on the cardinalities of *A* and *J*. The representation (24) shows that *k*-exclusion independence implies a special structure of the underlying interactions. It suggests to consider the family of functional modalities $(\kappa _J)$ given in terms of interactions $\phi _A$ with the structure

25 $$\begin{aligned} \phi _A (\varvec{x}_A, y) \; := \; \sum _{\begin{array}{c} J \subseteq A \\ |J| \le r \end{array}} \alpha _{A,J} \, \psi _J(\varvec{x}_J ; y) , \end{aligned}$$

where $\psi _J$ are arbitrary functions that do not depend on $x_i$, $i \notin J$. Note that these functions are not indexed by *A*. Given this structure, we have

26 $$\begin{aligned} \ln \kappa _J(\varvec{x}_J ; y)\sim & {} \sum _{A \subseteq J} \phi _A(\varvec{x}_A, y) \nonumber \\= & {} \sum _{A \subseteq J} \sum _{\begin{array}{c} J' \subseteq A \\ |J'| \le r \end{array}} \alpha _{A,J'} \, \psi _{J'}(\varvec{x}_{J'} ; y) \nonumber \\= & {} \sum _{\begin{array}{c} B \subseteq J \\ |B| \le r \end{array}} \beta _{J , B} \, \psi _{B}(\varvec{x}_{B} ; y), \end{aligned}$$

which represents a special case of (23). This proves that all functional modalities of a robust system involve interactions of order at most *r*. We denote by ${\mathcal M}_r$ the set of functional modalities $(\kappa _J)$ that have the representation (26). Note that in this representation, each $\kappa _J$ can have different coefficients $\beta _{J,B}$. More precisely, if $\psi _B$ appears in the representation of $\kappa _J$ and $\kappa _{J'}$, the corresponding coefficients $\beta _{J,B}$ and $\beta _{J',B}$ will be typically different. Summarising, we obtain the following result.

### Theorem 2

(see Theorem 15 in Rauh and Ay 2014) *Let* $(\kappa _J)$ *be a family of strictly positive functional modalities that is* *k*-*exclusion independent in all* $\varvec{x} \in {\mathcal {S}}$. *Then there exists a family* $(\kappa '_J)$ *of functional modalities in* ${\mathcal M}_r$, with $r = n - k$, *such that* $\kappa _J (\varvec{x} ; \cdot ) = \kappa '_J (\varvec{x} ; \cdot )$ *for all* $\varvec{x} \in {\mathcal {S}}$ *and all* $J \subseteq I$.

This result implies that, in order to achieve *k*-exclusion independence, the order of interaction should not be too large. Such an interaction will break down as result of a knockout so that the remaining units will not be able to compensate it. On the other hand, if the interactions are small enough, their removal can be compensated. This compensation is achieved by an appropriate change of the coefficients $\beta$ in the representation (26). To be more precise, say that the interaction $\psi _B$ has a coefficient $\beta _{I, B}$ in the unperturbed function $\kappa = \kappa _I$ and assume that the node set *K* is knocked out, where *B* is still a subset of the remaining set $J = I{\setminus } K$. Thus, in the post-knockout function $\kappa _J$, the term $\psi _B$ will still be involved. However, the coefficient $\beta _{J,B}$ will typically change in order to ensure the invariance of the function. Therefore, in order to design a robust system, we need the whole family of coefficients $\beta _{J, B}$. This would allow the system to react to a knockout by immediately switching to the right post-knockout function $\kappa _J$. Alternatively, the knockout might initiate a re-adaptation process, which takes time to recover the original function. In this case, it is not required to hard-wire all coefficients $\beta _{J,B}$ into the system. It is then sufficient to store the coefficients involved in the original function, that is $\beta _{I, B}$. The other coefficients are obtained in terms of the recovery process. Such a process is particularly relevant within the study of biological robustness mechanisms.

## Conclusions

We have reviewed results related to the robustness of function against knockout perturbations. In comparison with previous works, this article tries to draw a conceptually more complete picture, relying on existing theoretical results. On the one hand, it provides a solid justification of knockout experiments for system identification. On the other hand, it highlights the role of neutrality and low interaction order in the context of robust systems. These properties are associated with two corresponding characterisations of *k*-exclusion independent systems, Theorems 1 and 2. Thereby, neutrality and low interaction order, two at first sight seemingly unrelated aspects, are revealed to be equivalent. Even though we have this equivalence, we highlight an important difference in the corresponding representations. In the “neutrality representation” (8) the map $\kappa$ is only specified on the support ${\mathcal {S}}$ of the input distribution $\mu$. The output of the map $\kappa$ for configurations in the space between the connected components $N \in \mathfrak {N}({\mathcal {S}})$, on which $\kappa$ is constant, can be arbitrarily chosen. This is fundamentally different in the “interaction representation” (26) of $\kappa$, that is for $J = I$. It is indeed possible that the sum $$\sum _{\begin{subarray}{c} B \subseteq I \\ |B| \le r \end{subarray}} \beta _{I , B} \, \psi _{B}(\varvec{x}_{B} ; y)$$ is well-defined even for $\varvec{x} \notin {\mathcal {S}}$. The only requirement for $\varvec{x}$ is that for each restriction $\varvec{x}_B$ with $|B| \le r$, there is a configuration $\varvec{x}'_{I {\setminus } B}$ such that the concatenation $(\varvec{x}_B, \varvec{x}'_{I {\setminus } B})$ is in the support ${\mathcal {S}}$ of the input distribution. Thus, the “interaction representation” comes with an extrapolation of the values on the connected components $N \in \mathfrak {N}({\mathcal {S}})$ to some part of the space in between. The role of such an extrapolation for robustness is currently unclear. It might allow the system to generalise to and deal with unforeseen situations in a reasonable way. This is absolutely not possible with the “neutrality representation”, which is nothing but a lookup table for all $\varvec{x} \in {\mathcal {S}}$, with no restriction at all for $\varvec{x} \notin {\mathcal {S}}$. Coping with unforeseen situations in itself is a robustness property, which, however, is different from the way we have defined robustness in this article. Thus, the presented formalism might allow us to compare different notions of robustness. We already discussed one instance of this at the end of “Exclusion dependence” section: Invariance of function with respect to knockout perturbations implies invariance of function with respect to mutations.

## References

[CR1] Amari S (2016). Information geometry and its applications.

[CR2] Amari S, Nagaoka H (2000). Methods of information geometry.

[CR3] Ay N, Amari S (2015). A novel approach to canonical divergences within information geometry. Entropy.

[CR4] Ay N, Krakauer DC (2007). Geometric robustness theory and biological networks. Theory Biosci.

[CR5] Ay N, Flack J, Krakauer DC (2007). Robustness and complexity co-constructed in multimodal signalling networks. Philos Trans R Soc B Biol Sci.

[CR6] Ay N, Jost J, Vân Lê H, Schwachhöfer L (2017). Information geometry.

[CR7] Boldhaus G, Bertschinger N, Rauh J, Olbrich E, Klemm K (2010). Robustness of boolean dynamics under knockouts. Phys Rev E.

[CR8] de Visser JA, Hermisson J, Wagner GP, Meyers LA, Bagheri-Chaichian H, Blanchard JL, Chao L, Cheverud JM, Elena SF, Fontana W, Gibson G (2003). Perspective: evolution and detection of genetic robustness. Evolution.

[CR9] Flack JC, Girvan M, De Waal FB, Krakauer DC (2006). Policing stabilizes construction of social niches in primates. Nature.

[CR10] Fontana W (2006). The topology of the possible.

[CR11] Krakauer DC, Page KM, Erwin DH (2009). Diversity, dilemmas, and monopolies of niche construction. Am Nat.

[CR12] Krakauer DC, Flack J, Ay N (2010). Probabilistic design principles for robust multi-modal communication networks.

[CR13] Rauh J (2013). Generalized binomial edge ideals. Adv Appl Math.

[CR14] Rauh J, Ay N (2014). Robustness, canalyzing functions and systems design. Theory Biosci.

[CR15] Schuster P, Fontana W, Stadler PF, Hofacker IL (1994). From sequences to shapes and back: a case study in $$\text{RNA}$$ secondary structures. Proc R Soc Lond Ser B Biol Sci.

[CR16] Wagner A (2007). Robustness and evolvability in living systems.

